# Can a One-Time Subtle Attachment Security Priming Impact Outcomes in the Real World?

**DOI:** 10.3390/ijerph22030441

**Published:** 2025-03-17

**Authors:** Omri Gillath, Bruce S. Liese, Gery C. Karantzas

**Affiliations:** 1 Department of Psychology, University of Kansas, Lawrence, KS 66045, USA; 2 Department of Family Medicine, University of Kansas Medical Center, Kansas City, KS 66103, USA; bliese@kumc.edu; 3 School of Psychology, Faculty of Health, Melbourne Burwood Campus, Deakin University, Burwood, VIC 3125, Australia; gery.karantzas@deakin.edu.au

**Keywords:** attachment, security, priming, field study, helping

## Abstract

Although research exists on the impact of security priming, the vast majority of studies have been conducted in the laboratory and used repeated prime/priming sessions. The studies described in this paper test whether attachment security priming impacts people’s sense of security and related behaviors in the real world following a single exposure to a security prime. In the first two studies, participants were indirectly exposed to either security or control cues. In Study 1 (*n* = 53), exposure to security cues via posters near the entrance to the building where the study took place led to a higher sense of state security. In Study 2 (*n*~21,000), the same security primes (posters) led to a greater tendency to engage in helping behavior. In Study 3 (*n* = 200), exposure to similar security primes, embedded in a self-help guide, increased people’s positive evaluations of the health guide, which is known to be related to higher adherence to treatment. The implications for day-to-day security, well-being, and health are discussed.

## 1. Introduction

Psychological functioning, well-being, and health can be enhanced using simple laboratory-based priming methods designed to increase feelings of attachment security [1,2]. Can these lab-based effects be replicated in real-world settings? If so, is one-time exposure enough? Psychologists seek to understand, predict, and, when possible, influence human behavior. Priming is often used to change behavior in the laboratory. The successful application of priming methods outside the lab can go a long way toward fulfilling psychologists’ applied goals of positively shifting or changing people’s behavior and psychological well-being out in the real world [3]. The current research employs established laboratory methods to test the effects of attachment security priming in two real-world settings and assesses whether a subtle one-time exposure is enough to generate an effect on state attachment, helping, and the evaluation of health-related materials.

Priming affects people’s cognitions, emotions, and behavior, either with or without their awareness [4]. When performed in the laboratory, priming usually involves a participant’s exposure to different cues, one at a time, embedded in a carefully controlled environment. Researchers use such designs to *isolate* the cue of interest (i.e., the prime) spatially and temporally. These procedures, in turn, increase the chances that (1) the priming manipulation will be powerful enough to result in changes in cognitions or behaviors and (2) the obtained priming effects can be attributed to the cue rather than to a confounder. However, when using priming in a natural setting (we use the terms real-world and natural interchangeably)—like in a field study—participants are likely to be exposed to many other (distracting) cues alongside the prime. In such settings (complex, stimulus-rich environments), it is unknown whether priming effects are likely to occur, and especially whether single exposure to a prime is enough of a dosage to yield any effect.

Researchers who have used ecologically valid primes have taken a step toward examining priming in real-world environments. Such primes have been manipulated in ways that approximate real-life exposure to subtle cues—such as holding a hot cup of coffee [5], engaging with the American flag [6], or watching a soap opera on TV [7]. Some of these studies failed to replicate, while other studies demonstrated significant but not substantively large or long-lasting effects.

Only a small amount of research has been undertaken to investigate field priming within the attachment literature. The present research builds upon and extends this work by presenting ecologically valid attachment security primes in a natural setting. Existing field priming studies in attachment have mostly used repeated presentations of primes. However, the repeated priming of individuals has its challenges of participant attrition and ensuring that people are consistently exposed to the prime. Given these limitations, we aim to test the effects of a single priming dosage on various outcomes using ecologically valid primes. Positioning the primes in the field allows researchers to examine the effects of the prime within a real-world environment, with its restrictions and challenges to human processing.

In previous field priming studies, researchers either observed the way the environment affects cognitions, emotions, and behaviors (e.g., how cloudiness or the temperature affects these outcomes) or used components of the environment as experimental conditions (e.g., exposure to currency [8,9,10]; see also Paluck’s [7] work previously mentioned). Although priming methods have been previously applied to understand the functioning and underlying mechanisms of close relationships [11] and of adult attachment specifically, most of these studies were performed in the laboratory with undergraduate samples [12].

Baldwin and colleagues [13,14], who conducted some of the earliest priming work regarding attachment, suggest that attachment not only has a relatively stable trait-like aspect (attachment style) but a more transient aspect—state attachment [15,16]. Attachment styles—the way people think, feel, and behave in their relationships—involve individuals’ cognitive, affective, and behavioral patterns (or “modes”) in relationships [17,18]. These styles develop based on interactions with one’s primary caregivers during childhood and the internalization of these interactions by way of mental models. Individuals can have various models stored mentally (e.g., anxious, avoidant, secure). In hundreds of studies, attachment styles were found to be reliable predictors of relational behaviors and affect-regulation among adults [17,18].

State attachment is the most immediately accessible mental model, which may be in line with one’s disposition (or a general model [14]). However, people can momentarily behave differently than their dispositional attachment style as a function of the model that is most accessible at that point in time. Priming cues can change which model is more cognitively salient and accessible in a given moment. Indeed, laboratory studies have demonstrated that simple priming techniques can make people, at least temporarily, feel more or less secure [1].

In the current project, a novel approach to introducing priming cues was taken. The environment was manipulated to include subtle attachment security cues, intended to test the effects of these new cues on subject’s feelings, attitudes, and beliefs (for a similar approach, see work on advertisements [19]). In the first two studies, we tested the effects of security priming on two widely examined outcomes in the security priming literature—state attachment and prosocial behavior. In the third study, we introduced a novel extension of past research by priming patients in the waiting room of a medical clinic to increase positive reactions to a healthy eating self-help guide.

Using the same priming techniques in the field as those used in the laboratory enables researchers to maintain experimental control while increasing the validity and generalizability of previous priming effects. McGuire et al. [20] provided initial evidence to support the idea that the effects of attachment security priming can be generalized to and result in similar outcomes in the real world among a non-college population. They found that adolescents who were repeatedly exposed to security primes during their after-school activity showed lower depression symptoms than adolescents exposed to neutral primes. Carnelley et al. [21] showed similar findings with outpatients (lower depressed and anxious mood after security priming). Likewise, Otway et al. [22], after an initial priming task in the lab, used texting on three consecutive days to boost security. They found higher feelings of security right after the initial priming session and the day after the last prime. Conversely, Oehler and Psouni [23], using a similar priming procedure (visualization tasks via mobile texting), found no effect on state attachment security but did find reduced attachment avoidance (up to one week after the last priming). In all these studies, security priming was performed repeatedly and over time. Here, we test the impact of a one-time exposure to subtle attachment security primes in the real world.

### Current Studies

The aim of the current studies was to test whether a single exposure to attachment security priming, shown to be successful in a controlled laboratory environment, would have similar effects in the field. We focused on three outcomes—the sense of attachment security [15], the tendency to engage in prosocial behavior [24], and positive attitudes toward a self-help guide meant to increase healthy eating [25,26]. We hypothesized that security priming (compared to the control) would increase the sense of security (and potentially decrease insecurity), the tendency to help (increased willingness to participate in the study), and reporting positive attitudes regarding a healthy eating guide. Studies 1 and 2 were performed outside the laboratory, but still on campus, whereas Study 3 was conducted off campus in a medical center. Studies 2 and 3 targeted real-world behavior.

## 2. Study 1

The goal of Study 1 was to test whether individuals exposed to security cues in a real-world context would show an increase in their level of state security and a decrease in their level of state attachment insecurity. In line with past laboratory studies, we hypothesized that security priming will increase the sense of state attachment security and decrease the sense of state attachment anxiety and avoidance [1]. Study 1 (and Studies 2 and 3) was approved by the Institutional Review Board at the University of Kansas.

### 2.1. Method

#### 2.1.1. Participants

In total, 53 people (27 women, age 18–60 years, *Mdn* = 20) volunteered to participate in the study. Overall, 62 percent were Caucasian, 10% Asian, 12% Hispanic, 8% African American, and 8% were “other”; 94 percent reported themselves as heterosexual, and 68% were students.

#### 2.1.2. Materials and Procedure

Participants were recruited at the Student Union and the main hall of the dorms at a large North American university, mainly during the beginning of the semester. The experimenter placed posters with the prime pictures (see details below and pictures in the Supplementary Materials) near the building’s entrances. The posters were either located on a message board or on an easel next to the board. The secure and neutral posters were hung from Monday to Thursday (the weekend was not included), between 9 am and 5 pm, and the hours they were up were counterbalanced (that is, if on the first day the security poster was hung during a particular time period, on the next day, the neutral one was hung at that time). The Union and dorms were chosen as these places have high visitor traffic throughout the day. Both locations are relatively stimulus-rich environments (e.g., have many other posters hung), allowing the posters used as primes to blend in easily.

Posters were located at the entrance to the area where the table for the study was set up (~15 yards away from the table), such that students entering the building passed by them. Research assistants sitting or standing near the table asked passers-by to complete a short survey and offered them the chance to enter a raffle for a USD 20 bookstore voucher. Once participants agreed to take part and signed a consent form, they individually completed a battery of questionnaires, which was described as a study about the environment, personality, and well-being. Initial measures included state attachment (our main DV), self-esteem, mood, and demographic questions. After a distracter questionnaire, a trait attachment scale was completed, followed by self-esteem and mood scales. These scales were used as controls to rule out the possibility that the effects obtained were due to self-esteem or mood rather than attachment.

The security prime (*n* = 19) was a 28”-by-22” poster with a drawing by Picasso used in previous studies to prime attachment security [27]. In the drawing, a mother is embracing her baby while looking into his eyes. The drawing is in black and white and is thought to depict a core component of the secure-base schema or script—the receipt of care and comfort from a primary caregiver/attachment figure. The neutral prime (*n* = 29) was a poster of the same size that depicted an abstract drawing similar to the polygon used in Mikulincer et al. [27].

State attachment was measured using the State Adult Attachment Measure (SAAM) [15], a 21-item self-report instrument designed to assess current attachment-related anxiety, avoidance, and security. Participants were asked to think about the way they felt RIGHT NOW and rate the extent to which each item accurately described their feelings using a 7-point scale ranging from 1 (*not at all*) to 7 (*very much*). Each scale (anxiety (e.g., “I feel a strong need to be unconditionally loved right now”), avoidance (e.g., “If someone tried to get close to me, I would try to keep my distance”), and security (e.g., “I feel like I have someone to rely on”)) consisted of seven items (αs ranged from 0.83 to 0.90). Only the correlation between SAAM security and avoidance was significant (*r* = −0.61, *p* < 0.01).

Self-esteem was assessed using Rosenberg’s [28] 10-item self-esteem scale (e.g., “On the whole, I am satisfied with myself”.). Participants rated their agreement with each item on a 4-point scale ranging from 1 (*strongly disagree*) to 4 (*strongly agree*), with α = 0.84.

Mood was assessed using the *Positive and Negative Affect Schedule* (PANAS) [29], a 20-item measure (e.g., excited, nervous) that assesses how people feel right now. Participants rated the 20 adjectives using a 7-point scale from 1 (*very slightly or not at all*) to 7 (*extremely*), and the *α*s were 0.84 (positive) and 0.86 (negative).

To be able to assess whether the effects of priming occurred above and beyond one’s trait attachment, we used the short form of the Experiences in Close Relationships inventory (ECR-S) [30]. The ECR-S is a 12-item self-report instrument designed to measure dispositional attachment-related anxiety and avoidance. Participants were asked to think about their close relationships in general and rate the extent to which each item accurately described their feelings in these relationships, using a 7-point scale ranging from 1 (*not at all*) to 7 (*very much*). Each subscale (anxiety and avoidance) consists of six items, and the αs were 0.65 for avoidance and 0.71 for anxiety (the two scales were not correlated, *r* = 0.17, *ns*). Example items include “I need a lot of reassurance that I am loved by my partner” (anxiety) and “I am nervous when partners get too close to me” (avoidance).

At the end of the battery, participants were debriefed and questioned as to whether they had noticed the poster prime when they walked through the building. Nine participants noticed the posters (about 19% of our sample). In the analyses described below, this factor was controlled for, as previous work suggests that awareness of the prime may moderate its effectiveness [31]. This study was approved by the institutional review board of the first author’s university. Data were collected between 2008 and 2009.

### 2.2. Analysis Plan

Before analysis, variables were standardized, and outliers (±3 SDs from the mean) were dropped. The decision to drop outliers based on 3 SDs was decided a priori. One participant was dropped. No sensitivity tests were conducted. We used SPSS (version 28, IBM, Armonk, NY, USA) to run our analyses.

#### 2.2.1. Preliminary Analysis

To verify that the priming manipulation did not affect *trait* measures of attachment, or the other control measures, a MANOVA testing the effects of condition (attachment security vs. neutral) on trait attachment anxiety and avoidance, self-esteem, and positive and negative mood was conducted. As expected, no significant effects were revealed; all *Fs* < 1, *ns*.

#### 2.2.2. Main Analyses

Next, a correlational analysis (see Table 1a,b) and three hierarchical regression analyses (see Table 1c,d,e) were conducted to examine whether priming condition (attachment security vs. neutral) affects *state* security, anxiety, and avoidance. The first step of each regression included (centered) dispositional attachment anxiety and avoidance; the second step included self-esteem, positive and negative mood (to control for their potential effects), a dummy variable representing whether or not participants reported noticing the prime (not noticing was coded as 0), and sex. The third step included condition coded as a dummy variable (security coded as 1 and neutral coded as 0).

### 2.3. Results and Discussion

#### 2.3.1. State Attachment Security

As predicted, the analysis for state attachment security revealed a main effect for condition (secure vs. neutral), *β* = 0.31, *p* < 0.05, Δr^2^ = 0.08, such that the security prime led to higher scores on the state security measure. The analysis also revealed a main effect for attachment anxiety, *β* = −0.50, *p* < 0.001 (Δr^2^ = 0.31), where people higher on trait anxiety had a lower state security score.

#### 2.3.2. State Attachment Anxiety

The analysis predicting state attachment anxiety revealed only a main effect for negative affect, *β* = 0.42, *p* < 0.05, Δr^2^ = 0.24; thus, people who reported higher negative affect also reported higher state attachment anxiety. No other main effects were significant.

#### 2.3.3. State Attachment Avoidance

The analysis predicting state avoidance revealed main effects for trait attachment avoidance, *β* = 0.51, *p* < 0.001; trait attachment anxiety, *β* = 0.31, *p* < 0.05 (Δr^2^ for this step = 0.39); self-esteem, *β* = −0.42, *p* < 0.01; negative affect, *β* = 0.27, *p* < 0.05; and sex, *β* = −0.26, *p* < 0.05 (Δr^2^ for this step = 0.25). No other main effects were significant.

Overall, the results for Study 1 partially support our predictions. Exposing people to attachment security-related cues in natural (complex, stimulus-rich) settings enhanced people’s sense of attachment security but did not affect state attachment insecurity. This may have been the case because state security is easier to impact and requires weaker effects to change [1]. Our results are similar to those of McGuire et al. (2018) [20], Carnelley et al. (2018) [21], and Otway et al. (2014) [22], but they were obtained with a single dose. 

## 3. Study 2

The goal of Study 2 was to test whether security cues, which affected self-reported levels of attachment security in Study 1, would also affect behavior. We exposed people to the same cues as in Study 1 (security versus neutral primes) in a natural environment and then assessed behavioral changes. Specifically, we focused on prosociality, and measured people’s tendency to help others following the priming. Previous research has repeatedly demonstrated that security, both dispositional and manipulated, is associated with a higher tendency to help (e.g., tending to volunteer more [32]). The willingness to participate in a short study can be used to operationalize helping behavior (e.g., Fitzsimons & Bargh [33]). We hypothesized that security priming in a natural setting would have a similar effect to that found in laboratory studies, such that it would increase the tendency of people to help as measured in volunteering to complete the study’s battery. Unlike Study 1, participants were not promised any compensation for their time or effort. Any differences in the willingness to complete the battery were thought to reflect different prosocial behavioral tendencies.

### 3.1. Method

#### 3.1.1. Participants

In total 21,319 people passed by the study location; out of them, 82 (48 women, age 18–56 years, *Mdn* = 21; 44 were exposed to the secure prime, see Table 2) volunteered to participate in the study. In addition, 62% were Caucasian, 8% Asian, 10% Hispanic, 8% African American, and 12% were “other”; 88% reported themselves as heterosexual, and 63% were students (this was a new sample, not the same as in Study 1).

#### 3.1.2. Materials and Procedure

Participants were recruited at the Student Union and the main hall of the dorms at a large North American university. The arrangements of the table and the posters were the same as in Study 1 (28”-by-22” poster at the entrance to the building). However, unlike Study 1, participants were not offered participation in a raffle as compensation for their time. Two research assistants (RAs, blind to the purpose of the study and the meaning of the conditions) oversaw each session. One RA used a clicker to count people passing by (every pass was counted, even if the same person crossed more than once). The other RA offered passersby the chance to participate in a study without telling them what the study was about or promising any compensation. Once a participant agreed to partake and signed a consent form, they completed a demographic questionnaire. The main dependent variable was agreeing to help the experimenter by participating in the study. The study was approved by the institutional review board of the first author’s university. Data were collected between 2010 and 2011.

### 3.2. Analysis Plan

Before the analysis, variables were standardized, and outliers ±3 SDs from the mean were dropped. No participants were dropped. We used SPSS (version 28) to run our analyses. To analyze whether the number of people who volunteered to help was significantly different in the experimental group (security prime) compared with the control group (abstract/neutral prime), Pearson’s Chi-squared test with Yates’ continuity correction was used.

### 3.3. Results and Discussion

The analysis revealed a significant effect for prime type, χ^2^ (df = 1) = 4.60, *p* < 0.05, such that more people stopped to help in the experimental condition compared to the control one.

Overall, Study 2 provided further support to the claim that security priming would have similar effects in the field to those obtained in the laboratory. Furthermore, Study 2 provided behavioral evidence that goes beyond self-report assessments and extends to outcomes beyond state attachment security and insecurity to prosociality.

## 4. Study 3

The goal of Study 3 was to test whether security cues, which affected self-reported levels of attachment security, and prosociality, would also affect people’s attitudes toward healthy behavior advice. To achieve this, we exposed people via brochures to either attachment security or neutral primes in a natural environment (waiting area at the hospital) and then assessed their evaluations of health messaging—specifically healthy eating behavior. Previous research has demonstrated the possibility of changing eating habits using a simple self-help handbook and Adapted Motivational Interviewing (AMI) [34,35]. In other research, attachment security priming was shown to improve attitudes toward a range of therapies [26]. Furthermore, attachment security was found to predict higher adherence to treatment [25]. In line with these findings, we tested here whether using security priming embedded within a self-help guide would be associated with positive evaluations of the guide. We hypothesized that one-time exposure to a subtle attachment security prime in a natural setting (hospital outpatient waiting area) would have a similar effect to that found in laboratory studies, such that it would be associated with more positive attitudes toward a healthy eating guide compared to a control prime.

### 4.1. Method

#### 4.1.1. Participants

A new sample totaling 200 participants (aged 18–84 years, *Mdn* = 48, 143 women) participated in the study. Overall, 54%were Caucasian, 23% African American, 12% Hispanic, 4.5% Asian, 1.5% Native American, and the rest chose “mixed” or “other”. In addition, 7% had a high school education, 22% had a high school education, 19% had some college education, 5% were current college students, 29% had a college degree, and 21% had a higher degree. Furthermore, 9 percent were married, 6% were not married but in a serious, committed relationship, 15% were cohabiting (all these participants were coded as in a relationship), 59% were single, and the rest did not report. The average BMI was 29.97 (range 17.22–65.37), and on average, people exercised three days a week.

#### 4.1.2. Materials and Procedure

The research took place in a major university medical center in North America. On any given day, there may be as many as 20–30 patients in a waiting room before seeing their family physicians for a wide range of acute and chronic medical conditions. Because it was a family medicine clinic, patients represented various ages, socioeconomic backgrounds, family circumstances, and body mass index (BMI) measures. It was these patients that we recruited for the study while they were in the waiting area. Sixty to seventy percent of Americans are obese or overweight [36]. Furthermore, many patients with chronic medical problems are obese or overweight [37]. Hence, the patients in this family medicine clinic were considered an appropriate group for a study involving an eating habit guide.

Four research assistants (RAs; all undergraduate psychology students) were trained to approach patients and ask about their interest in this study. Those who expressed an interest and consented were assigned to one of two experimental groups: secure or control. The research assistants were blinded to the purpose of the study and the meaning of the conditions. Participants then completed background measures, went through the self-help guide with the RA, and were offered a chance to keep the guide. Next, participants completed various self-report questionnaires, including ratings of the self-help guide and demographics.

##### Self-Help Guide

All participants received a 21-page self-help *Healthy Eating Guide* based on the book *Taming the Hungry Bear: Your Way to Recover from Chronic Overeating* [38]. This *Healthy Eating Guide* has been shown to effect positive changes in binge eaters [34]. The guide contains educational techniques in the following eight sections:

1. What is unhealthy eating?
2. Learning to take small steps
3. Understanding hunger and food cravings
4. Beginning the work
5. Working with hunger and appetite
6. Working with food and feelings
7. Preventing relapse
8. Local mental health and Internet resources.

The original guide has images of fruits and vegetables throughout. We replaced these images with either attachment-security-related images or neutral ones. Half of the participants (*n* = 100) received a guide containing attachment security images. These images portrayed individuals being in close, warm, nurturing contact with other individuals (friends, family members, etc.). The other participants (*n* = 100) received the same *Healthy Eating Guide* but one containing neutral images—images of individuals in more formal encounters (meetings, asking for directions, etc.).

##### Ratings of the Self-Help Guide

Participants were asked how interesting and easy to understand the guide was, how comforting the pictures were (representing attachment security), and how much they liked the guide. This study was approved by the institutional review board of the first author’s university. Data were collected between 2016–2017.

### 4.2. Analysis Plan

Before analysis, variables were standardized, and outliers ±3 SDs from the mean were dropped. No participants were dropped. We used SPSS (version 28) to run our analyses. Because Study 3’s participants reflected a different (older and more diverse) population to Studies 1 and 2, we included age, gender, and relationship status, in addition to the type of prime (secure/neutral), as predictors in our analysis. We used a regression analysis to examine whether prime type (attachment security coded as 1 vs. neutral coded as 0) influenced people’s views of the self-help guide. We first calculated a total score from the means of understanding, liking, finding it interesting, and evaluating the pictures as comforting (α = 0.72, see means and SDs in Table 3). We then ran a regression analysis with all the predictors entered in one step.

### 4.3. Results and Discussion

The regression revealed a main effect for prime type, *β* = 0.17, *p* = 0.020, and a main effect for relationship status, *β* = −0.17, *p* = 0.020 (see Table 4). The results of Study 3 provided further support to the proposition that one-time exposure to security priming has an impact in the field. Specifically, we demonstrated that security priming resulted in people showing more interest in and understanding of the self-help guide, liking it more, and evaluating the pictures as more comforting.

## 5. General Discussion

The three studies reported here provide evidence to support the effects of a subtle one-time security priming on people’s sense of security. While there are ample studies on security priming in the lab, and even some on priming outside the lab, there is a grave need for more field research, especially studies that test whether subtle and one-time security priming (as is often the case when exposed to such cues in the real world) can impact theoretically relevant outcomes. Our studies provide evidence that people exposed to security priming in the field, feel more secure, are more likely to engage in prosocial behavior, and evaluate healthy eating advice in the form of a self-help guide more positively. Importantly, these results were obtained in complex stimulus-rich real-world settings. As such, they bear important theoretical and practical implications.

### 5.1. Theoretical Implications

Our environment is heavily loaded with cues and stimuli. A single cue, and especially one that is not salient (e.g., not very big, colorful, or noisy), is therefore less likely to be noticed. Despite that, exposure to our simple black-and-white pictures/posters affected people’s sense of security and their tendency to help, in line with our theory-driven predictions. This suggests that compared with other non-attachment-related cues (e.g., the abstract poster), people may be more sensitive to social and especially attachment-related cues in the environment. In accordance with Bowlby’s [39] conceptualization, it seems that people are hard-wired to detect stimuli related to security and insecurity and readily react to such cues.

Research has already shown the importance of warning signs for survival and well-being (e.g., a buzzer before the cage’s floor is being electrified or a siren warning before a missile attack; for a review, see Maren & Quirk [40]). As important, or even more important, are the signals to relax (e.g., a different sound or light that no electric shock would be applied, or a siren letting people know the missile attack had passed), allowing the organism to return to its routine existence or a baseline, with lower stress, and a chance to recover [41]. In the case of attachment, such signs serve not only to signal that safety and security have been regained but also that exploration (as well as other non-attachment-related behaviors) can continue. This is highly important for survival (e.g., exploring the environment increases the chances of finding new resources like water and food). With the increase in loneliness [42], people’s sensitivity to social signs of safety and security might increase [43], making our findings even more important.

Finding security effects, even when controlling for self-esteem and positive affect, fits previous work, demonstrating the unique effects of security enhancement [15,27]. Mikulincer et al. [27] showed that a security prime but not a positive affect prime buffers against threat and negative cues. In the current studies, security primes maintained their unique qualities outside the laboratory. In other words, briefly passing the poster prime seems to provide something beyond a mere boost to self-esteem or an increase in positive mood. Likewise, skimming a brochure or a flyer with secure images could potentially help with better treatment outcomes and increased adherence. Future studies will have to test these possibilities.

### 5.2. Practical Implications

The current studies suggest that priming methods that were tested in the laboratory may be usefully applied in public contexts (like schools or hospitals), boosting people’s sense of security and, in turn, extending the beneficial effects of security beyond the laboratory. For instance, posters used in schools or road signs along highways might lower the levels of aggression and increase tolerance and prosocial tendencies [44,45]. However, before these priming methods could be applied more broadly, further testing should take place. Replicating the current findings, perhaps in a different context or with variations on the priming methods used in this research, would provide additional information on making this priming more efficient and better controlled.

### 5.3. Limitations

First, the security prime in each study was only compared to a neutral prime, which leaves the possibility that the effects were due to cues of affiliation or social interactions or merely the exposure to human figures (but see Study 3). That said, previous studies on security priming have repeatedly demonstrated the unique effects of security priming compared with cues of positive affect, social interactions, or exposure to human figures that are not one’s attachment figures [24]. This study’s main goal was to demonstrate that security priming would benefit people’s sense of security, tendency to help **outside the laboratory**, and increase positive attitudes toward a healthy behavior guide, not to compare its effects to other primes. Future studies should expand the current findings by examining the effects of other potential contextual cues and comparing them to the effects of attachment security cues.

Another limitation relates to the extent of the effects. Based on the current results, it is unclear how long the priming effects last and whether or not they are likely to affect other outcomes. This should also be examined in future studies where the dependent measures would apply to how people may help others in need, such as the unhoused or those who are marginalized, and the enactment of health behaviors, such as adhering to health advice. Such studies should also go beyond the one-time security priming and include repeated priming [20], which is likely to have stronger effects.

A third limitation is related to the lack of manipulation checks in Studies 2 and 3. Although similar results were obtained in laboratory studies, future studies should potentially include a manipulation check to verify that the priming worked (similar to the results of Study 1). Likewise, future studies should try and replicate our findings in non-WEIRD samples while using alternative methods and scales (e.g., a different security measure) to increase generalizability.

## 6. Conclusions

Despite these limitations, the current set of studies provides coherent and consistent results that bridge lab and fieldwork in the attachment priming literature. All three studies used a similar methodology—exposure to attachment-security-related images (via posters in Studies 1 and 2 and a self-help guide in Study 3). The methodology is simple, cheap, and flexible, making it highly accessible and easily integrated into other interventions. The studies’ results generally replicated existing in-lab results on attachment security priming (for a review, see Gillath et al. [1]). Going beyond existing work, our results confirm the ability of a one-time exposure to security priming to generate beneficial effects in real life. The studies contribute to the understanding of attachment processes and how they operate in different contexts and locations [46] and contribute to the proliferation of scientific work from the laboratory to the field. The findings also extend the understanding of how people process their environment and how exposure to certain cues affects humans’ cognition and behaviors. Finally, this set of innovative findings infuses a sense of hope that a simple application, such as putting secure pictures into dorms or hospitals, might help people feel more secure, less defensive, and more open to others and experiences in line with the *Broaden and Build* model of attachment [24,46,47].

## Figures and Tables

**Table 1:** (**a**) Correlations between main variables (Study 1). (**b**) Ranges, means, and SDs for the SAAM subscales (Study 1). (**c**) Regression predicting SAAM security. (**d**) Regression predicting SAAM anxiety. (**e**) Regression predicting SAAM avoidance.

(a)									
		2	3	4	5	6	7	8	9
SAAMsec		0.004	−0.57 **	−0.27	−0.53 **	0.32	−0.29	0.37 **	0.04
2. SAAManx			−0.05	−0.06	0.26	−0.02	0.40	−0.14	−0.04
3. SAAMavo				0.53 **	0.36 *	−0.18	0.51 **	−0.53 **	0.08
4. Avoidance					0.08	−0.14	0.44 **	−0.12	0.11
5. Anxiety						−0.20	0.38 *	−0.21	0.21
6. PA							−0.15	0.42	−0.19
7. NA								−0.16	0.18
8. Self-esteem									0.18
9. Prime									
(**b**)									
**Condition**		**N**		**Range**		**Mean**		**Std. Deviation**	
1	SAAMsec	29		5.00		5.6010		1.29215	
	SAAManx	29		4.71		4.4581		1.19118	
	SAAMavo	29		5.00		2.5714		1.18850	
	Valid N (listwise)	29							
2	SAAMsec	19		2.71		5.7218		0.97761	
	SAAManx	19		4.14		4.2782		1.09366	
	SAAMavo	19		4.57		2.8120		1.27691	
	Valid N (listwise)	19							
(**c**)									
**Model**		**Unstandardized Coefficients**		**Standardized Coefficients**		**t**	**Sig.**	**95.0% Confidence Interval for B**	
		**B**	**Std. Error**	**Beta**				**Lower Bound**	**Upper Bound**
1	(Constant)	5.710	0.152			37.560	<0.001	5.403	6.018
	Z_AVOIDANCE	−0.232	0.156	−0.200		−1.491	0.144	−0.546	0.083
	Z_ANXIETY	−0.576	0.155	−0.498		−3.717	<0.001	−0.890	−0.263
2	(Constant)	4.979	1.285			3.874	<0.001	2.367	7.591
	Z_AVOIDANCE	−0.176	0.174	−0.152		−1.013	0.318	−0.530	0.177
	Z_ANXIETY	−0.443	0.178	−0.383		−2.487	0.018	−0.805	−0.081
	Notice	−0.430	0.423	−0.148		−1.018	0.316	−1.289	0.429
	Sex	0.736	0.328	0.321		2.242	0.032	0.069	1.404
	Self-esteem	−0.312	0.313	−0.145		−0.999	0.325	−0.948	0.323
	Positive affect	0.148	0.225	0.096		0.655	0.517	−0.310	0.606
	Negative affect	−0.087	0.266	−0.053		−0.328	0.745	−0.629	0.454
3	(Constant)	4.925	1.210			4.070	<0.001	2.463	7.387
	Z_AVOIDANCE	−0.207	0.164	−0.178		−1.261	0.216	−0.542	0.127
	Z_ANXIETY	−0.489	0.169	−0.422		−2.893	0.007	−0.832	−0.145
	Notice	−0.737	0.419	−0.254		−1.759	0.088	−1.590	0.116
	Sex	0.826	0.312	0.360		2.651	0.012	0.192	1.460
	Self-esteem	−0.396	0.297	−0.183		−1.335	0.191	−0.999	0.208
	Positive affect	0.144	0.212	0.093		0.677	0.503	−0.288	0.576
	Negative affect	−0.145	0.252	−0.088		−0.576	0.568	−0.658	0.368
	Prime	0.752	0.325	0.313		2.317	0.027	0.092	1.412
(**d**)									
**Model**		**Unstandardized Coefficients**		**Standardized Coefficients**			**Sig.**	**95.0% Confidence Interval for B**	
		**B**	**Std. Error**	**Beta**				**Lower Bound**	**Upper Bound**
1	(Constant)	4.297	0.192			22.387	<0.001	3.909	4.685
	Z_AVOIDANCE	−0.044	0.196	−0.035		−0.226	0.822	−0.442	0.353
	Z_ANXIETY	0.328	0.196	0.261		1.675	0.102	−0.068	0.724
2	(Constant)	0.951	1.520			0.626	0.536	−2.138	4.040
	Z_AVOIDANCE	−0.323	0.206	−0.256		−1.567	0.126	−0.741	0.096
	Z_ANXIETY	0.202	0.211	0.161		0.960	0.344	−0.226	0.630
	Notice	−0.728	0.500	−0.231		−1.458	0.154	−1.744	0.287
	Sex	0.232	0.388	0.093		0.599	0.553	−0.557	1.022
	Self-esteem	0.380	0.370	0.162		1.026	0.312	−0.372	1.131
	Positive affect	0.309	0.267	0.184		1.158	0.255	−0.233	0.851
	Negative affect	0.766	0.315	0.425		2.430	0.021	0.125	1.406
3	(Constant)	0.963	1.539			0.626	0.536	−2.169	4.095
	Z_AVOIDANCE	−0.316	0.209	−0.250		−1.510	0.141	−0.741	0.110
	Z_ANXIETY	0.212	0.215	0.169		0.988	0.330	−0.225	0.649
	Notice	−0.660	0.533	−0.209		−1.238	0.225	−1.745	0.425
	Sex	0.213	0.396	0.085		0.537	0.595	−0.594	1.019
	Self-esteem	0.398	0.377	0.170		1.055	0.299	−0.370	1.166
	Positive affect	0.310	0.270	0.184		1.147	0.260	−0.240	0.859
	Negative affect	0.779	0.321	0.433		2.428	0.021	0.126	1.431
	Prime	−0.167	0.413	−0.064		−0.404	0.689	−1.007	0.673
(**e**)									
**Model**		**Unstandardized Coefficients**		**Standardized Coefficients**		**t**	**Sig.**	**95.0% Confidence Interval for B**	
		**B**	**Std. Error**	**Beta**				**Lower Bound**	**Upper Bound**
1	(Constant)	2.639	0.152			17.377	<0.001	2.332	2.946
	Z_AVOIDANCE	0.634	0.155	0.512		4.080	<0.001	0.319	0.948
	Z_ANXIETY	0.385	0.155	0.312		2.483	0.017	0.071	0.698
2	(Constant)	0.553	1.080			0.512	0.612	−1.641	2.747
	Z_AVOIDANCE	0.441	0.146	0.357		3.018	0.005	0.144	0.739
	Z_ANXIETY	0.129	0.150	0.104		0.859	0.396	−0.175	0.433
	Notice	0.356	0.355	0.115		1.004	0.322	−0.365	1.078
	Sex	−0.639	0.276	−0.262		−2.318	0.027	−1.200	−0.079
	Self-esteem	0.975	0.263	0.424		3.712	<0.001	0.441	1.509
	Positive affect	0.070	0.189	0.042		0.370	0.714	−0.315	0.455
	Negative affect	0.477	0.224	0.270		2.130	0.040	0.022	0.932
3	(Constant)	0.574	1.079			0.532	0.598	−1.622	2.769
	Z_AVOIDANCE	0.453	0.147	0.366		3.092	0.004	0.155	0.752
	Z_ANXIETY	0.146	0.151	0.119		0.972	0.338	−0.160	0.453
	Notice	0.477	0.374	0.154		1.276	0.211	−0.284	1.238
	Sex	−0.674	0.278	−0.276		−2.428	0.021	−1.240	−0.109
	Self-esteem	1.008	0.265	0.438		3.811	<0.001	0.470	1.546
	Positive affect	0.072	0.189	0.043		0.378	0.708	−0.313	0.457
	Negative affect	0.500	0.225	0.283		2.222	0.033	0.042	0.957
	Prime	−0.295	0.289	−0.115		−1.020	0.315	−0.884	0.294

* *p* < 0.05; ** *p* < 0.01.

**Table 2:** Number of participants who passed by and stopped (Study 2).

	Control	Security
Passed by	12,501	8818
Stopped	38	44

**Table 3:** Means, SDs, and ranges (Study 3).

Prime		N	Minimum	Maximum	Mean	Std. Deviation
neutral	Total_score	100	0.00	4.00	3.3350	0.73273
	Valid N (listwise)	100				
secure	Total_Score	100	1.75	4.00	3.4992	0.46428
	Valid N (listwise)	100				

**Table 4:** Regression analysis predicting attitudes toward the self-help guide (Study 3).

Model		Unstandardized Coefficients		Standardized Coefficients	t	Sig.	95.0% Confidence Interval for B	
		B	Std. Error	Beta			Lower Bound	Upper Bound
1	Constant	3.658	0.167		21.855	<0.001	3.328	3.989
	prime	0.207	0.088	0.167	2.341	0.020	0.033	0.381
	age	−0.004	0.003	−0.109	−1.475	0.142	−0.009	0.001
	gender	−0.041	0.103	−0.029	−0.394	0.694	−0.243	0.162
	in_rel	−0.210	0.089	−0.168	−2.344	0.020	−0.386	−0.033

## Data Availability

The original contributions presented in this study are included in the article/Supplementary Materials. Further inquiries can be directed to the corresponding author.

## Supplementary Materials

The following supporting information can be downloaded at https://www.mdpi.com/article/10.3390/ijerph22030441/s1. supplementary File S1: posters with the prime pictures.
