# Supplementary figures and images for: Can a One-Time Subtle Attachment Security Priming Impact Outcomes in the Real World?
Source: Int J Environ Res Public Health. 2025 Mar 17;22(3):441. doi: 10.3390/ijerph22030441 (PMC11942572; doi:10.3390/ijerph22030441)

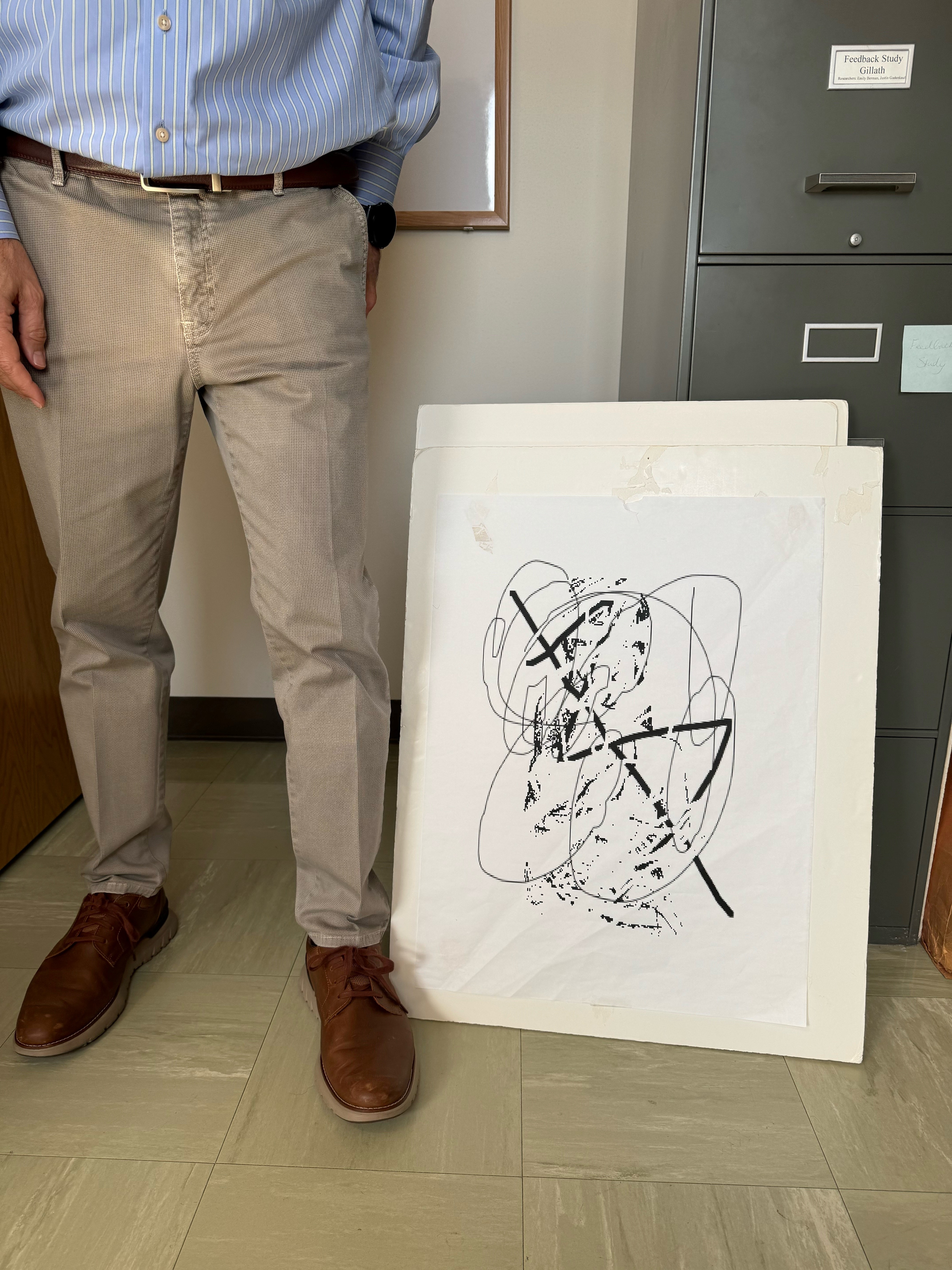

Supplement: Supplementary file 1 [file ijerph-22-00441-s001.zip › Neutral prime poster.jpg]

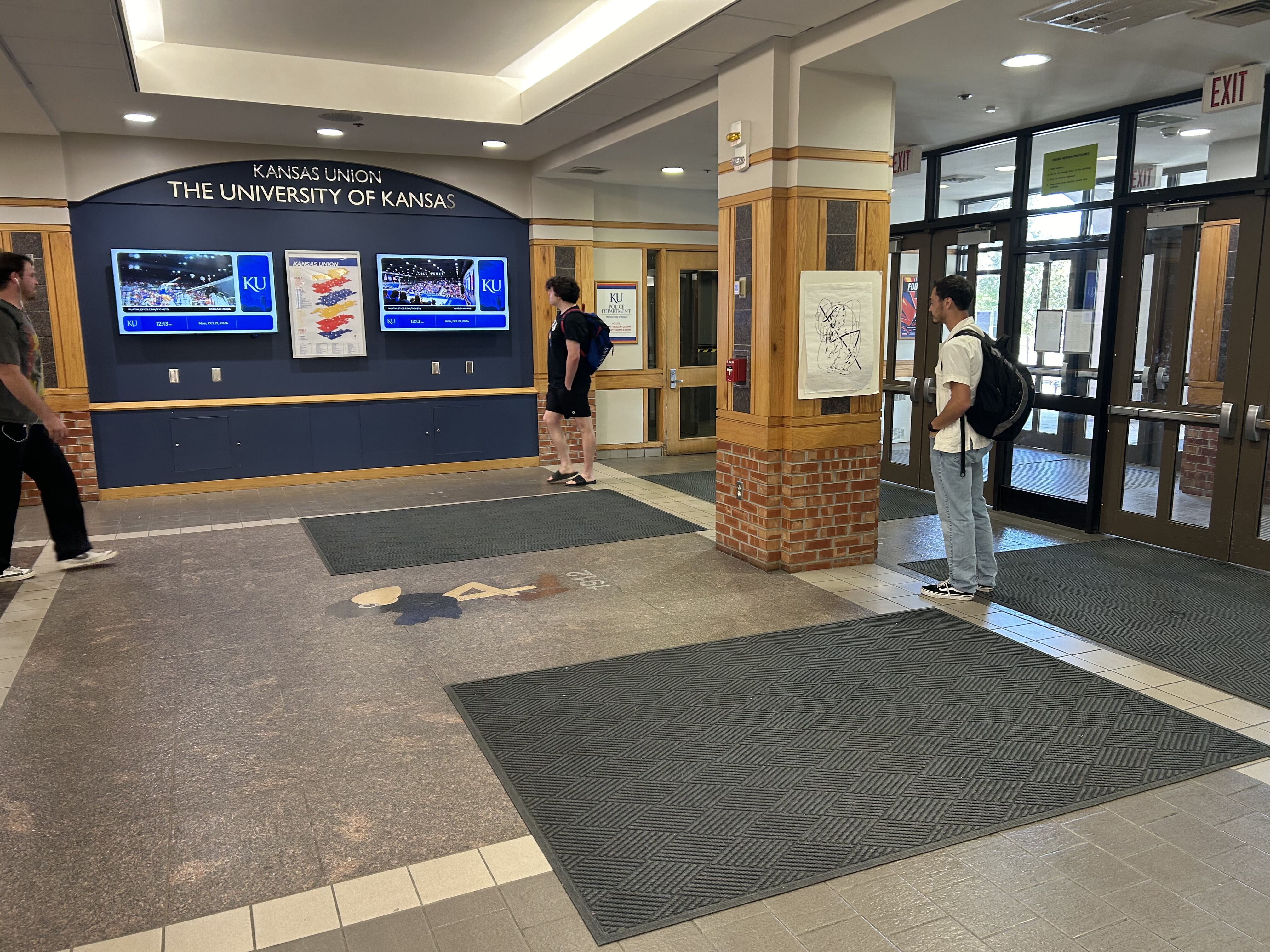

Supplement: Supplementary file 1 [file ijerph-22-00441-s001.zip › Neutral prime union entrance.jpg]

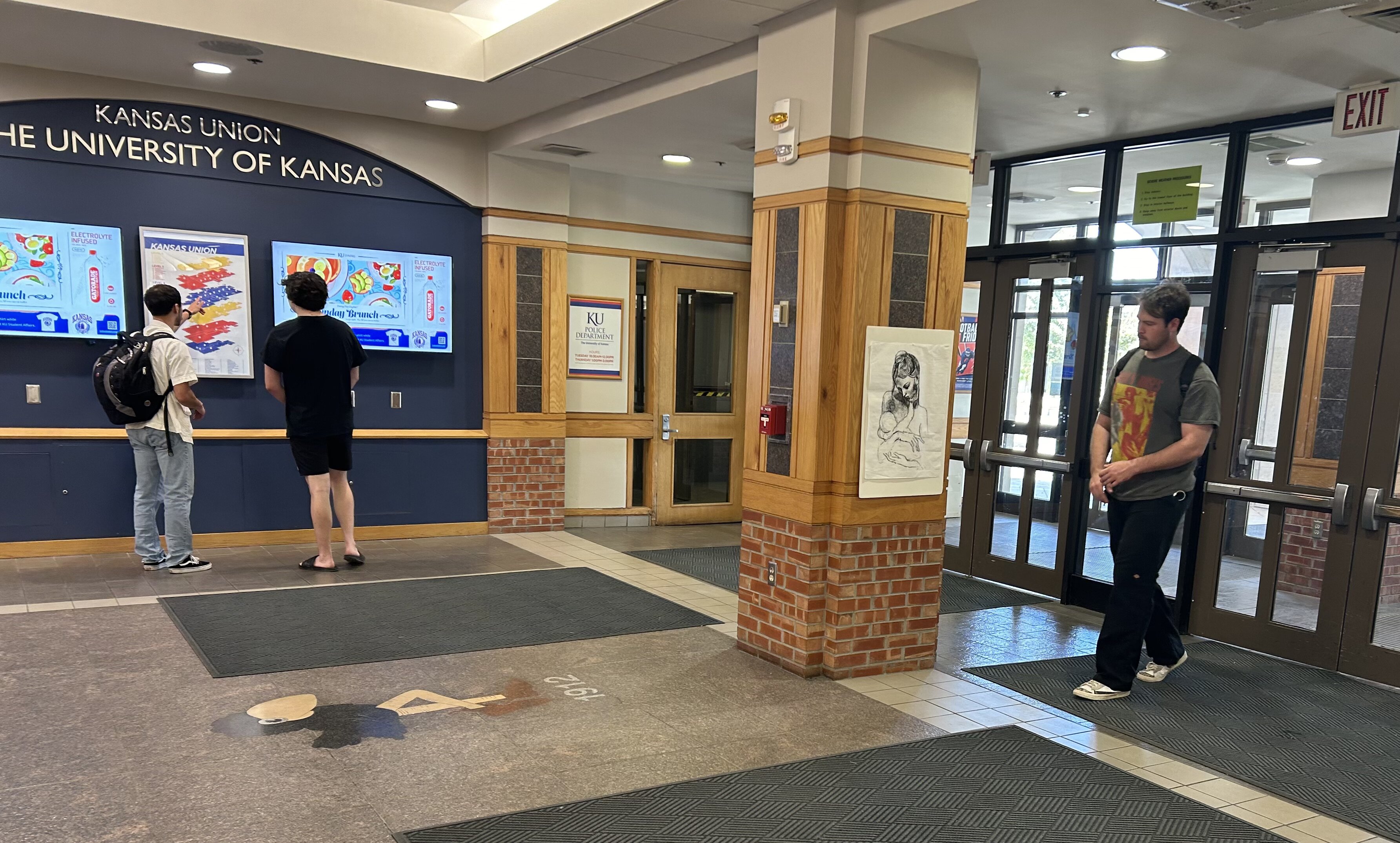

Supplement: Supplementary file 1 [file ijerph-22-00441-s001.zip › Secure prime union entrance.jpg]

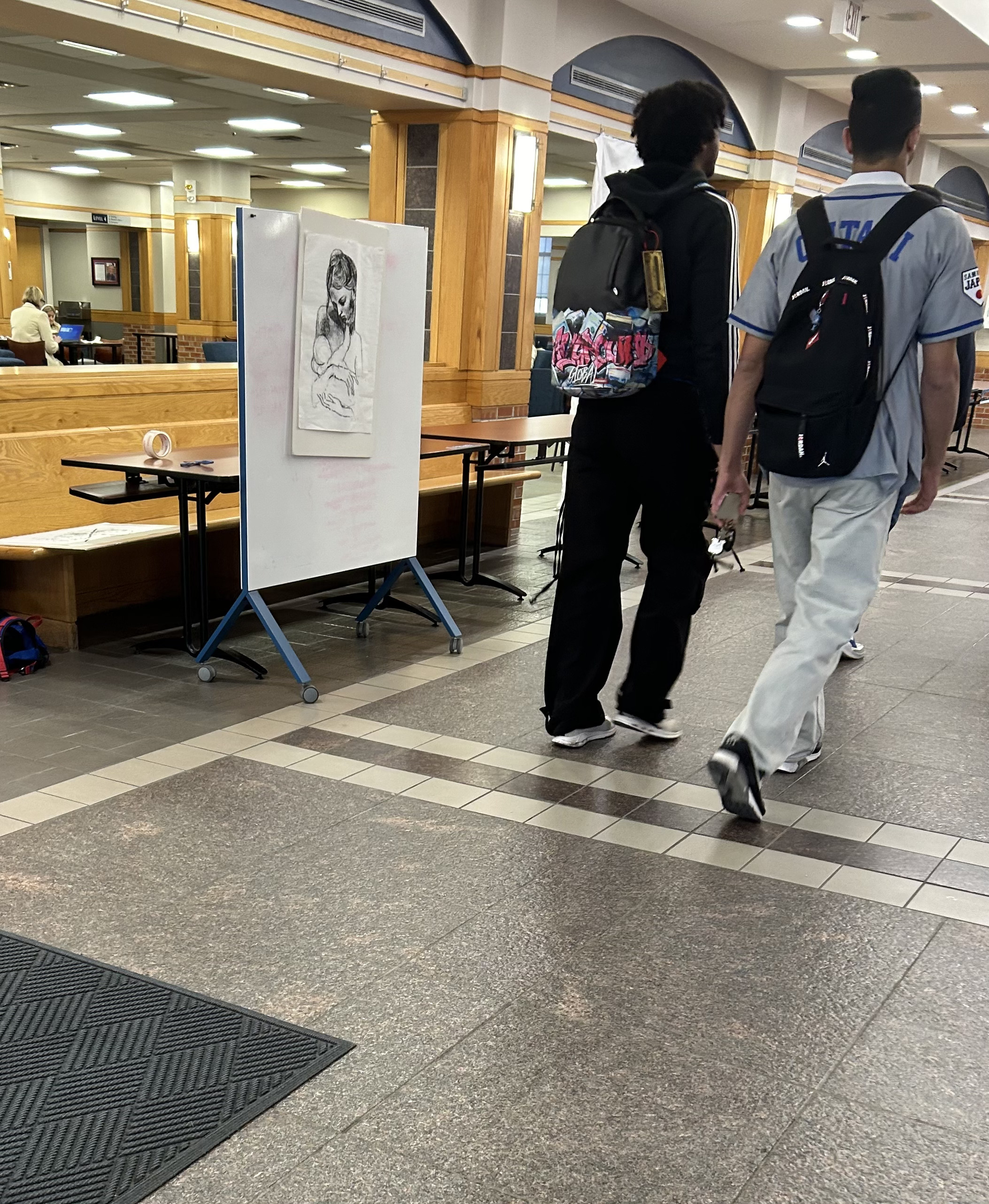

Supplement: Supplementary file 1 [file ijerph-22-00441-s001.zip › Secure prime union entrance1.jpg]

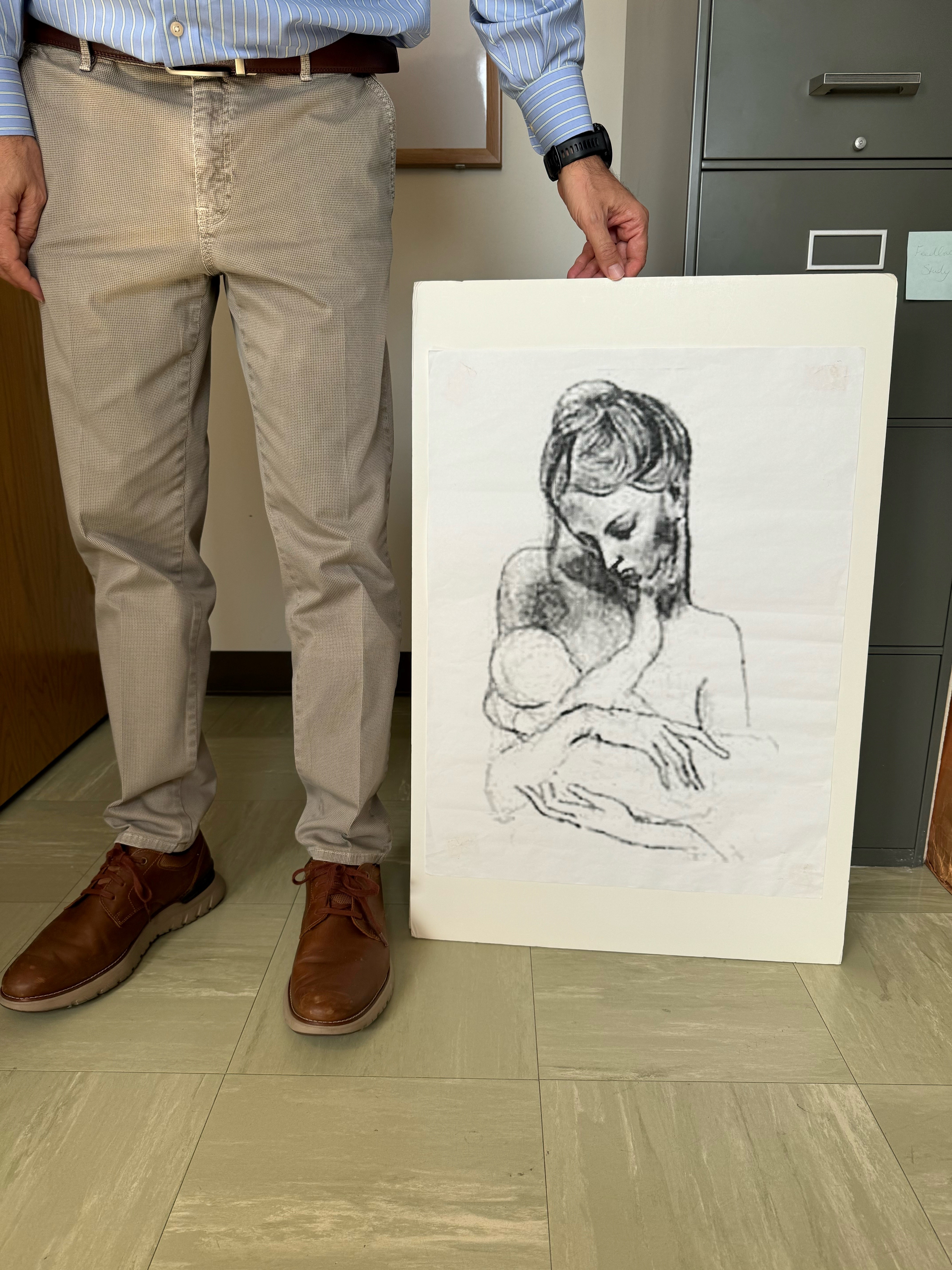

Supplement: Supplementary file 1 [file ijerph-22-00441-s001.zip › Security prime poster.jpg]
